# Racing to Normal: A Case Report of Rapid and Notable Cholesterol Reduction With Statin and Ezetimibe Combined Therapy

**DOI:** 10.7759/cureus.80004

**Published:** 2025-03-03

**Authors:** Daniel Aparício, Marta Costa, Rui Marques

**Affiliations:** 1 Internal Medicine, Unidade Local de Saúde Viseu Dão-Lafões, Viseu, PRT

**Keywords:** dyslipidemia, ezetimibe+statin, lipids and stroke, low-fat diet, risk factors for cardiovascular diseases

## Abstract

Severe mixed dyslipidemia is a major risk factor for cardiovascular disease (CVD), necessitating early and intensive lipid-lowering interventions. This case report describes the management of a 40-year-old patient with extreme dyslipidemia and high cardiovascular risk, emphasizing the impact of pharmacological therapy and lifestyle modifications.

Initial therapy included rosuvastatin 20 mg, ezetimibe 10 mg, and fenofibrate 145 mg, combined with smoking cessation, alcohol restriction, and dietary changes. The combined therapy resulted in progressive improvement, with final lipid levels at 16 months showing total cholesterol of 95 mg/dL, low-density lipoprotein cholesterol (LDL-C) of 11.7 mg/dL, high-density lipoprotein cholesterol (HDL-C) of 28.2 mg/dL, and triglycerides of 275 mg/dL. Echocardiographic and vascular studies confirmed atherosclerosis without significant luminal stenosis. Genetic testing for familial hypercholesterolemia was negative.

This case highlights the effectiveness of early combination therapy and lifestyle modification in managing severe dyslipidemia. The substantial lipid reduction achieved reinforces current recommendations for early and aggressive treatment in high-risk patients. Continuous monitoring and individualized therapy adjustments remain essential for long-term cardiovascular risk reduction.

## Introduction

Cardiovascular disease (CVD) remains the leading cause of mortality worldwide, accounting for an estimated 18 million deaths in 2019. A broad spectrum of cardiovascular conditions, including heart disease, cerebrovascular disease, and chronic kidney disease, are associated with increased morbidity and mortality. Alarmingly, only 18% of men with this spectrum of CVD have their condition adequately controlled [1].

Hypertension and non-high-density lipoprotein (HDL) cholesterol (HDL-C) are two of the most significant contributors to cardiovascular events, playing a crucial role in disease progression [2]. Elevated low-density lipoprotein (LDL) cholesterol (LDL-C) is recognized as a key modifiable risk factor within the cardiovascular continuum [3], and its management is essential in reducing the incidence of adverse outcomes such as stroke, acute myocardial infarction, coronary artery disease, and cardiovascular death [4].

Despite the availability of effective lipid-lowering therapies, numerous studies indicate that more than half of patients in Portugal fail to achieve optimal lipid control [5]. The SANTORINI study demonstrated that a combination therapy of statins and ezetimibe is more effective in reaching LDL-C target levels compared to statin monotherapy, highlighting the potential benefits of a dual therapeutic approach [6].

Current clinical guidelines emphasize the importance of early identification of cardiovascular risk factors and prompt initiation of appropriate treatment strategies. The Systematic Coronary Risk Evaluation 2 (SCORE2) risk assessment charts enable clinicians to stratify patients according to their cardiovascular risk and establish individualized treatment targets [7]. Given the synergistic effect of statins and ezetimibe, combination therapy is increasingly recognized as a preferred strategy for achieving optimal lipid control and reducing cardiovascular risk, having an average LDL reduction effect of 18% in combination, instead of the 6% obtained by doubling the statin dose [8].

## Case presentation

Here, we describe a case of a 40-year-old man, autonomous and professionally active but with a sedentary lifestyle and no regular physical exercise, who was previously healthy, with no known medical conditions or family history of dyslipidemia, and was referred to a hospital consultation for dyslipidemia and cardiovascular risk assessment due to analytical changes in the lipid profile. He had no regular medication but reported an active smoking history of 20 pack-years, an erratic diet with regular alcohol consumption, and a high-fat dietary pattern.

Routine laboratory tests revealed a fasting total cholesterol level of 634 mg/dL, an HDL-C level of 93.2 mg/dL, and triglycerides that could not be calculated. During the initial consultation, his body mass index (BMI) was within the normal range (23.4 kg/m²), and home blood pressure readings were consistently below 130 mmHg (systolic) and 90 mmHg (diastolic), ruling out hypertension. His cardiovascular risk, assessed using the SCORE2, was 39.6%, indicating a high 10-year risk of CVD [9].

Between the first and second consultations (at two months), repeat laboratory testing confirmed the initial results. A metabolic workup showed normal renal function, a uric acid level of 7.0 mg/dL, and a glycated hemoglobin (HbA1c) level of 5.7% with fasting blood glucose of 102 mg/dL. Combination therapy was initiated with rosuvastatin 20 mg and ezetimibe 10 mg in a single daily pill, along with fenofibrate 145 mg once daily. The patient was also advised to quit smoking, limit alcohol intake close to abstinence, adopt a low-fat diet, and engage in regular physical activity aiming for at least 150 minutes of aerobic exercise per week at moderate intensity.

At the three-month follow-up visit, after maintaining the prescribed medication and implementing lifestyle changes, total cholesterol had significantly decreased to 124 mg/dL, HDL-C to 24.9 mg/dL, and triglycerides to 647.7 mg/dL, with LDL-C remaining incalculable. The therapeutic regimen was adjusted by doubling the previous fenofibrate dose and adding cholestyramine (4 g with main meals).

At the 12-month follow-up, total cholesterol was 167 mg/dL, HDL-C was 29.1 mg/dL, and triglycerides were 1,281 mg/dL. Due to the patient’s thrombotic risk, clopidogrel 75 mg once daily was introduced.

By the 16-month follow-up, lipid levels had further improved, with total cholesterol at 95 mg/dL, LDL-C at 11.7 mg/dL, HDL-C at 28.2 mg/dL, and triglycerides at 275 mg/dL. Table 1 summarizes the evolution of cholesterol and triglyceride levels.

**Table 1:** Evolution of Laboratory Results in Mixed Dyslipidemia Study LDL-C: low-density lipoprotein cholesterol; HDL-C: high-density lipoprotein cholesterol

Time point	Total cholesterol (mg/dL)	LDL-C (mg/dL)	HDL-C (mg/dL)	Triglycerides (mg/dL)
Baseline	634	N/A	93.2	Not calculable
3 months	124	N/A	24.9	647.7
12 months	167	N/A	29.1	1,281
16 months	95	11.7	28.2	275

Additional investigations, including a transthoracic echocardiogram, ruled out morphological or functional abnormalities. A carotid and cervical arterial ultrasound revealed bilateral homogeneous atheromatosis with mild generalized calcification, preserved luminal patency, and no significant Doppler alterations.

This case highlights a patient with severely elevated cholesterol and triglyceride levels, whose cardiovascular risk was significantly reduced through a combination of lifestyle modifications and pharmacological intervention.

The patient continues follow-up in a hospital clinic with regular biochemical monitoring, cardiovascular risk assessment, and additional investigations for associated conditions (such as the assessment of hypertension, renal function abnormalities, and morphofunctional changes in the heart), maintaining the therapy instituted at the 12-month visit. Genetic testing for familial hypercholesterolemia was negative, and testing for familial hypertriglyceridemia is pending.

## Discussion

This case report highlights the management of a 40-year-old patient with severe dyslipidemia, demonstrating a remarkable response to combined lipid-lowering therapy and lifestyle modifications. The initial laboratory results indicated extreme hypercholesterolemia with a total cholesterol level of 634 mg/dL and a non-calculable LDL-C due to significantly elevated triglycerides. Despite the absence of a family history of dyslipidemia, the lipid profile suggested a potential underlying genetic component or secondary causes related to lifestyle habits, including active smoking, high alcohol consumption, and a hyperlipidemic diet.

The cardiovascular risk assessment using SCORE2 placed the patient at an extremely high risk, emphasizing the necessity for immediate and aggressive intervention [9]. Initial treatment with rosuvastatin 20 mg and ezetimibe 10 mg in a single-pill combination was mandatory regarding the risk, aligned with current guidelines, which advocate for early combination therapy in high-risk patients to maximize lipid-lowering efficacy and reduce long-term cardiovascular risk [7].

Despite the remarkable lipid-lowering response, the persistent elevation in triglycerides highlights the complexity of managing mixed dyslipidemia. Factors such as dietary adherence, alcohol consumption, and potential metabolic contributions may have played a role in the slower normalization of triglyceride levels. The introduction of clopidogrel at 12 months was justified by the patient’s continued atherogenic profile and high thrombotic risk.

Further investigations, including echocardiography and vascular imaging, confirmed the presence of generalized atherosclerosis with preserved luminal integrity, reinforcing the necessity for long-term lipid control to prevent cardiovascular events. The negative genetic testing for familial hypercholesterolemia suggests that the extreme lipid profile may have been predominantly lifestyle-driven rather than monogenic in origin.

This case exemplifies the critical role of early and intensive lipid-lowering therapy in high-risk patients. The impressive response to treatment demonstrates the efficacy of combined pharmacological strategies, particularly in individuals with extreme baseline lipid levels. Additionally, it reinforces the importance of lifestyle modifications, including smoking cessation, dietary changes, and physical activity, as integral components of dyslipidemia management [7].

Current guidelines advocate for a shift toward combination lipid-lowering therapy earlier in treatment algorithms, particularly in patients with severe dyslipidemia and high cardiovascular risk. The synergistic effects of statins, ezetimibe, and fibrates, as observed in this case, support this approach and highlight the need for personalized treatment strategies to achieve optimal lipid targets [7].

Long-term follow-up is essential to ensure continued lipid control and cardiovascular risk reduction. Ongoing monitoring, adherence assessment, and potential additional therapeutic adjustments will be key in maintaining the favorable outcomes achieved in this patient. This case reinforces the importance of a proactive and aggressive treatment approach to dyslipidemia, aligning with evolving clinical guidelines and evidence-based practice.

## Conclusions

This case report underscores the importance of early identification and aggressive management of severe dyslipidemia in high-risk patients. The reduction in lipid levels achieved through a combination of pharmacological intervention and lifestyle modifications demonstrates the efficacy of a structured treatment approach. The case highlights the need for early combination therapy, as supported by current guidelines, to maximize lipid-lowering effects and reduce long-term cardiovascular risk. The persistence of elevated triglycerides despite substantial LDL-C reduction and without changes in the glycemic profile emphasizes the complexity of dyslipidemia management and the necessity for ongoing follow-up and treatment optimization. This case reinforces the critical role of personalized, guideline-driven strategies in achieving optimal lipid control and preventing cardiovascular events.
